# Daily cost of consumer food wasted, inedible, and consumed in the United States, 2001–2016

**DOI:** 10.1186/s12937-020-00552-w

**Published:** 2020-04-20

**Authors:** Zach Conrad

**Affiliations:** Department of Health Sciences, William & Mary, Williamsburg, Virginia 23185 USA

**Keywords:** Food waste, Cost, Expenditure, Sustainability, NHANES, FoodAPS, LAFA

## Abstract

**Background:**

Consumer food waste in the United States represents substantial amounts of wasted nutrients, as well as needless environmental impact from wasted agricultural inputs, energy use, and greenhouse gas emissions. Efforts to reduce food waste at the consumer level are urgently needed to address the most prominent nutrition and environmental sustainability issues we now face. Importantly, individuals report that saving money is a salient motivator for reducing food waste, yet contemporary evidence on the consumer cost of wasted food is lacking. The objectives of this study are to 1) estimate the daily per capita cost of food wasted, inedible, and consumed 2) at home and away from home, and 3) by food group.

**Methods:**

This study utilizes cross-sectional, nationally-representative data on food intake from the National Health and Nutrition Examination Survey (2001–2016), linked with nationally representative data on food waste from published literature, as well as data on food prices and food price inflation from multiple publicly-available sources. Survey-weighted procedures estimated daily per capita expenditure on food waste for 39,758 adults aged ≥20 y.

**Results:**

Total daily per capita food expenditure was $13.27, representing 27% wasted, 14% inedible, and 59% consumed. The greatest daily food waste expenditures were observed for meat and seafood purchased for consumption outside of the home ($0.94, 95% CI: $0.90–0.99), and fruits and vegetables purchased for consumption in the home ($0.68, $0.63–0.73).

**Conclusions:**

The most cost-effective ways to reduce food waste at the consumer level are to focus waste reduction efforts on meat and seafood purchased for consumption outside of the home and fruits and vegetables purchased for consumption in the home. A number of strategies are available to help consumers reduce their food waste, which can increase their financial flexibility to purchase more healthy foods while simultaneously reducing environmental impact.

## Background

Diet quality in the United States remains far below recommended levels [1–3]. Less than 10% of Americans consume the recommended amounts of fruits and vegetables, and 60–70% exceed the recommendations for empty calories like added sugars and saturated fat [2]. Poor diet is now the leading risk factor for morbidity and mortality in the US, accounting for over 14% of disability-adjusted life-years and over 500 thousand deaths per year [4, 5].

At the same time, the average American wastes about one pound of food every day, including large amounts of healthy foods like fruits and vegetables [6]. This represents enough micronutrients like fiber, calcium, and vitamin D to close the gaps between actual and recommended intakes for tens of millions of people [7]. Food waste also represents massive amounts of wasted agricultural inputs like pesticides, fertilizers, irrigation water, and energy, and contributes to environmental problems like greenhouse gas emissions, water pollution, soil erosion, and biodiversity loss [8, 9].

Efforts to reduce food waste at the consumer level are urgently needed to help address the most prominent nutrition and environmental sustainability issues in the US and globally. Importantly, many Americans report that saving money is the most salient motivator for reducing food waste [10]. Along with continued public health messaging and clinical counseling, these savings could in turn be used to purchase more healthy foods.

Buzby and Hyman [11] and Venkat [12] have estimated that consumer food waste accounted for $1.07/day in 2008 and $1.10/day in 2009–2011, respectively. However, given limitations on the availability of food price data at the time, these studies assumed that all foods were purchased for at-home consumption (e.g., at supermarkets and grocery stores), which does not account for the important price difference compared to foods that are purchased for consumption away from home (e.g., at restaurants and vending machines) [13]. These studies also relied on food availability data as a proxy for food intake, which do not provide information on all foods consumed by individuals on a daily basis [14]. Since that time, advanced methods of estimating consumer food waste have emerged, which allow for the estimation of inter-individual variability in consumer food waste for all foods reported consumed on a daily basis [6]. Also since that time, a novel, nationally-representative consumer survey allows for the estimation of daily food expenditures for food consumed at home and food consumed away from home [15].

There is an urgent need to better understand the daily cost of consumer food waste in contemporary dollars, and to account for the important difference in food prices between foods purchased for at-home consumption and those purchased for consumption away from home. This information is needed to better understand opportunities to target consumer reductions in food waste so that individuals can have greater financial flexibility to purchase healthier foods while also reducing environmental impact. To address these important research gaps, the objectives of this study are to 1) estimate the daily per capita cost of food wasted, inedible, and consumed 2) at home and away from home, and 3) by food group.

## Methods

### Food consumption data

Individual-level data on food intake and sociodemography were acquired from the National Health and Nutrition Examination Survey (NHANES) 2001–2016 from 39,758 individuals ≥20 y [16]. NHANES is a cross-sectional, multi-stage, continuous survey maintained by the National Center for Health Statistics. Data are collected from approximately 5000 individuals per year and released on a two-year cycle. Dietary data are collected from each individual using a 24-h recall administered by a trained interviewer using United States Department of Agriculture’s (USDA) Automated Multiple Pass Method [17], and a subset of the study population completes a subsequent 24-h recall by telephone on a non-consecutive day. Only data from day 1 were used because this represents per capita intake [18]. As part of the dietary recall procedure, individuals provided information on whether foods were consumed at home (FAH) or away from home (FAFH).

### Data on food waste and inedible portions

NHANES provides data on foods as they were reported consumed, which is often in the form of dishes with multiple ingredients. Each NHANES food was disaggregated into its component ingredients using the Food Commodity Intake Database (FCID, 2001–2010), which provides data on the weight of nearly 500 ingredients included in each NHANES dish [19]. Each ingredient was subsequently linked with a distinct food commodity (i.e., ingredient) in the USDA Loss-adjusted Food Availability data series (LAFA), which provides estimates of waste and inedible portions for over 200 commodities [14]. Finally, all ingredients were re-aggregated to derive the amount of each NHANES dish wasted, inedible, and consumed. The details of this procedure are described elsewhere [6, 20] and depicted in Supplemental Figure 1. Sources of uncertainty and embedded assumptions are described in Additional file 1.

### Food price data

**Fig. 1:**
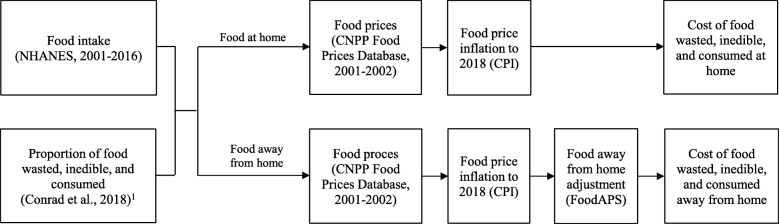
Methodology for estimating the cost of food wasted, inedible, and consumed at home and away from home. ^1^Conrad, Zach; Niles, Meredith; Neher, Deb; Roy, Eric; Tichenor, Nicole; Jahns, Lisa. (2018). Relationship between diet quality, food waste, and environmental sustainability. *PLoS ONE,* 13:e0195405. CNPP: Center for Nutrition Policy and Promotion, US Department of Agriculture. CPI: Consumer Price Index. LAFA: Loss-adjusted Food Availability data series. NHANES: National Health and Nutrition Examination Survey. FoodAPS: National Household Food Acquisition and Purchase Survey

Data on the price of each NHANES food were derived using multiple sources, as depicted in Fig. 1. Retail price for each NHANES food was obtained from USDA Center for Nutrition Policy and Promotion (CNPP) Food Prices Database, 2001–2002. CNPP Food Prices Database presents prices for foods acquired from supermarkets, grocery stores, convenience stores, supercenters, farmers’ markets, and other food stores for at-home consumption. Missing food prices (12%) were computed by averaging the individual food prices in each unique food category (*n* = 41 categories; Supplemental Table 1), weighted by the consumption amount (from NHANES) of each food within each food category. CNPP Food Prices Database does not include prices for alcoholic beverages, so these data were not included in the present study.

CNPP Food Prices Database provides only retail prices (FAH prices), so the price of each FAFH was derived using data from the National Household Food Acquisition and Purchase Survey (FoodAPS; Fig. 1) [15]. FoodAPS is a cross-sectional, multi-stage survey maintained by USDA Economic Research Service, and provides data on the price of FAH and FAFH collected from scanned barcodes and food receipts from a sample of 4305 households [15]. Using FoodAPS data, a coefficient was derived that represents the ratio between the price paid for each FAH to the price paid for each FAFH, for each major food group (Supplemental Table 2). This coefficient was then multiplied by the price of each FAFH in the CNPP Food Prices Database to derive its adjusted price. For example, if the price of a given FAFH vegetable was $1.75 (from CNPP Food Prices Database), and if the average price of FAFH vegetables was 1.53 times greater than the average price of FAH vegetables (from FoodAPS), then the adjusted price would be $2.68 ($1.75 × 1.53). To make these findings relevant to contemporary economic conditions, all food prices were further adjusted to their 2016 US dollar value using the Consumer Price Index (CPI) specific to each major food group (Supplemental Table 3) [21]. The CPI represents the average change in consumer prices over time, and is maintained and published by the US Department of Labor, Bureau of Labor Statistics [21].

### Main analysis

The difference between the daily per capita expenditure on food wasted, inedible, and consumed from FAH and FAFH, as well as by food group, was tested using paired Wald tests. Statistical significance was set at *P* < 0.05. All analyses were adjusted for the multi-stage sampling design and survey weights of NHANES and FoodAPS using standardized procedures and variables [22, 23]. Stata 15.1 (StataCorp; College Station, TX) was used for data management and analyses.

### Sensitivity analysis

To provide a check on the robusticity of the assumptions embedded in the main analysis, several sensitivity analyses were conducted to investigate various sources of uncertainty. The uncertainty of food price data (inflation and FAFH estimates) was assessed by comparing the main results to three sensitivity models: Model 1 used 2008 food prices, Model 2 used FAH prices, and Model 3 represented a composite of Model 1 and Model 2 (2008 food prices and FAH prices). Uncertainty in food intake data was assessed by comparing the main results to two additional sensitivity models. Specifically, these models were needed to investigate whether the use of FCID to disaggregate NHANES foods into their component ingredients resulted in biased estimates of food intake over time, since FCID has not been updated since 2010 and may not be applicable to NHANES surveys after that date. Separate analyses were conducted for two distinct time points, 2001–2010 (Model 4) and 2011–2016 (Model 5). The main analysis was tested against each of the sensitivity models using Wald tests with *P* < 0.05 and Bonferroni adjustment for multiple comparisons.

## Results

The analytic sample included 39,758 individuals ≥20 y (Table 1). Over two-thirds (68%) were 31–70 years of age, and over one-half (52%) were female. Nearly 80% of individuals were non-Hispanic white. The majority (61%) of individuals either attended some college or graduated college, and nearly two-thirds (65%) had an income-to-poverty ratio of at least 2.00.

**Table 1:** Characteristics of study population, 2001–2016

Characteristic	n^a^	Percent (95% CI)^b^	
Age (y)	39,758		
20–30		21.2	(20.2–22.2)
31–50		37.5	(36.5–38.6)
51–70		30.4	(29.4–31.3)
70+		10.9	(10.4–11.5)
Sex	39,758		
Women		51.9	(51.3–52.4)
Men		48.1	(47.6–48.7)
Race/ethnicity	33,341		
Non-Hispanic white		77.9	(75.7–80.0)
Non-Hispanic black		12.7	(11.3–14.3)
Mexican-American		9.3	(8.0–10.8)
Education	39,719		
Less than high school		17.2	(16.2–18.2)
High school or equivalent		23.4	(22.5–24.3)
Some college		31.6	(30.7–32.4)
College graduate		27.8	(26.3–29.4)
Income-to-poverty ratio	36,784		
< 0.75		9.1	(8.3–9.8)
0.75–1.30		13.0	(12.2–13.8)
1.31–1.99		13.3	(12.6–13.9)
2.00–3.99		28.8	(27.7–29.8)
4.00+		35.9	(34.3–37.6)

^a^Sample sizes are unweighted

^b^Percentages within each column adjusted for survey weight

**Fig. 2:**
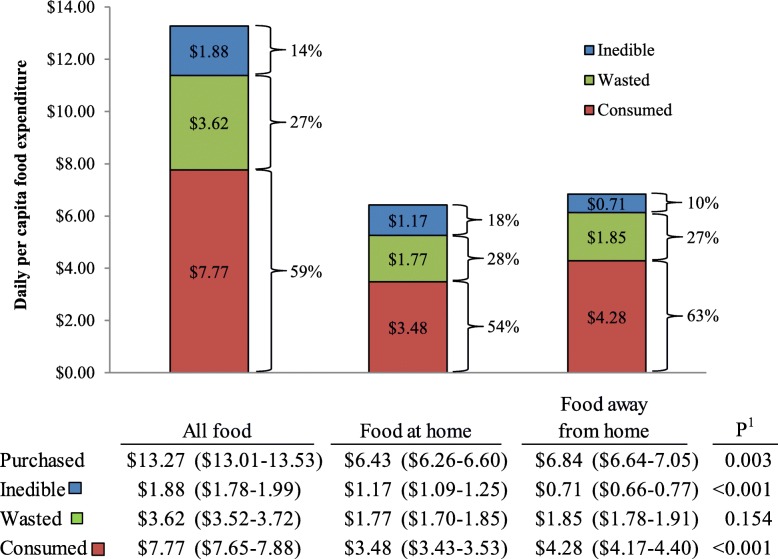
Daily per capita cost of food purchased, inedible, wasted, and consumed 2001–2016 (*n* = 39,758). Food purchased = wasted + inedible + consumed. ^1^Paired Wald test used to estimate the difference between food at home and food away from home (*P* < 0.05)

Total daily per capita food expenditure was $13.27 (95% CI: $13.01–13.53), representing 27% wasted ($7.77, $7.65–7.88), 14% inedible ($3.62, $3.52–3.72), and 59% consumed ($1.88, $1.78–1.99; Fig. 2). FAH accounted for 48% of food purchased (wasted + inedible + consumed), representing $6.43 ($6.26–6.60); and FAFH accounted for 52% of food purchased, representing $6.84 ($6.64–7.05). FAFH expenditures were greater than FAH expenditures for food purchased (*P* = 0.003), inedible (*P* < 0.001), and consumed (*P* < 0.001), yet no difference (*P* = 0.154) was observed between expenditures on wasted food between FAH ($1.77, $1.70–1.85) and FAFH ($1.85, $1.78–1.91).

**Fig. 3:**
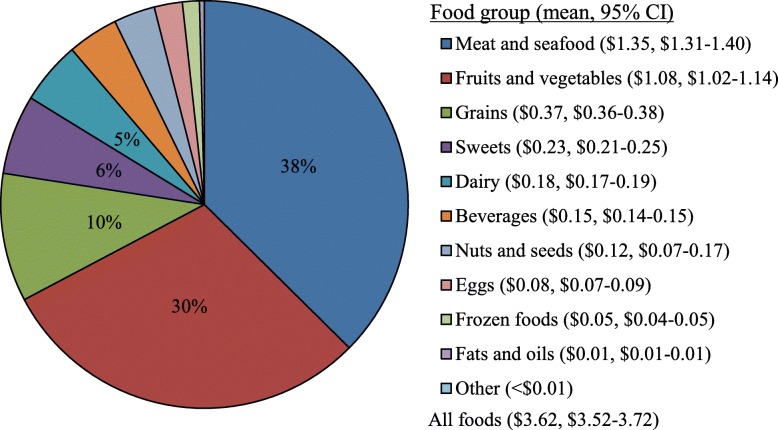
Daily per capita cost of total food waste by food group, 2001–2016 (n = 39,758). The following food groups each represent < 5% of total daily cost of food waste: dairy, beverages, nuts and seeds, eggs, frozen foods, fats and oils, and other foods. 95% CI not displayed for food groups with <$0.01 daily waste. The following foods represent the predominant ingredient in mixed dishes: fruits and vegetables, meat and seafood, grains, sweets, dairy, and eggs

Meat and seafood represented the greatest daily expenditure on food waste (38%), followed by fruits and vegetables (30%), grains (10%), sweets (6%), and dairy (5%); beverages, nuts and seeds, eggs, frozen foods, fats and oils, and other foods each represented less than 5% (Fig. 3). Compared to FAH wasted, greater expenditures on FAFH wasted were observed for meat and seafood, and eggs; and greater expenditures for FAH wasted were observed for fruits and vegetables, grains, dairy, frozen and shelf-stable foods, nuts and seeds, and nonalcoholic beverages (*P* < 0.05 for all comparisons; Supplemental Table 4).

Sensitivity analyses demonstrated that using 2008 food prices (Model 1), FAH food prices (Model 2), and a combination of both (Model 3) resulted in a decrease in food waste expenditures by $0.36, $0.64, and $1.04, respectively (*P* < 0.05 for all comparisons). Separate sensitivity analyses demonstrated no difference (*P* = 0.071) in food waste expenditure when restricting the food intake data to 2001–2010 (Model 4), and a decrease of $0.30 (*P* = 0.006) when restricting the food intake data to 2011–2016 (Model 5).

## Discussion

This is the first study, to our knowledge, to estimate the daily per capita cost of food wasted, inedible, and consumed in the US from 2001 to 2016. This study integrated dietary data from nearly 40 thousand adults collected over a 16-year period with nationally-representative data on food waste, food prices, eating location, and food price inflation.

We demonstrate that consumers spent, on average, over one-quarter of their daily food budget on food that ended up being wasted, representing over $3.50 per day. Meat and seafood accounted for the greatest proportion of daily food budgets spent on wasted food, followed by fruits and vegetables, grains, sweets, and dairy. No difference in total food waste expenditure was observed between food consumed at home (FAH) and food consumed away from home (FAFH), but greater expenditure was observed for FAFH meat and seafood and FAH fruits and vegetables.

Others have estimated that daily per capita food waste at the consumer level represented approximately $1.07–1.10 [11, 12], which is lower than estimates presented here ($3.62). However, the estimates from previous studies were derived by applying FAH prices from 2008 to 2009 to all foods reported consumed, which does not account for the important price differences between FAH (e.g., at supermarkets and grocery stores) and FAFH (e.g., at restaurants and vending machines), and does not account for food price inflation that has occurred since that time. Sensitivity analyses revealed that accounting for these differences reduced food price estimates in the present study to $2.58. The remaining gap between the present results and previous studies is likely due to differences in data coverage in the underlying datasets, where NHANES (used in the present study) provides a complete accounting of individual-level data on all foods reported consumed, whereas LAFA (used in previous studies) provides population-level data on the amount of select food commodities available for consumption. In the present study, established methods were utilized to incorporate the strengths of both datasets buy linking them together using FCID, which allowed for an estimation of the amount of waste attributable to each ingredient in each food consumed by each individual.

According to recently-updated food expenditure estimates published by the US Department of Agriculture [24], the average household food expenditure in 2016 was $30.18 [25] (1.95 adults per household [26]=$15.48 per adult), representing 52% of FAH and 48% FAFH [25], which is consistent with our findings (food expenditure was $13.27 per adult, not including alcohol, representing 48% FAH and 52% FAFH). Others have estimated that, using 2001–2004 FAH prices, mean expenditure on consumed food was $4.81 [27] and $5.79 (per 2000 kcal) [28], which straddles our estimate of $5.21 (Model 3).

The present study suggests that the average American consumer spends over $1300 per year ($3.62 per day × 365 days) on food that ends up being wasted, which is greater than the annual expenditure on vehicle gasoline ($1250); apparel ($1207); household heating and electricity ($1149); property taxes ($1046); and household maintenance, repairs, and insurance ($936) for the average single-person household in 2017 [29]. Since individuals report that saving money is the most important motivator for reducing food waste [10], contextualizing the cost of food waste within other common household expenditures can be one avenue to encourage behavior change. Realistically, it may not be possible to eliminate all consumer food waste because households (especially those with children) face competing demands on time, diverse food preferences, and other practical considerations [30], and discarding spoiled food is a key aspect of ensuring appropriate food safety standards in the household. But targeted efforts to reduce food waste can help individuals and households make positive changes toward increasing their food budgets and reducing environmental impact.

The present study demonstrates that meat and seafood, as well as fruits and vegetables, accounted for the greatest value of consumer food waste by food group, cumulatively representing over 60% of the total, which is consistent with previous findings [11, 12]. Importantly, our novel method for distinguishing food waste in the home versus outside of the home yields important new information that can be used by consumers to better target reductions in food waste: the greatest amount of meat and seafood waste (by value) occurred outside of the home ($0.41 in the home vs. $0.94 outside of the home), whereas the greatest amount of fruit and vegetable waste occurred within the home ($0.68 in the home vs. $0.40 outside of the home). Several strategies are available to help consumers navigate these complexities. For example, when eating meat and seafood dishes prepared outside of the home (at restaurants, for example), consumers can strive to match their hunger level with meal portion size by sharing meals and ordering smaller portions [31, 32], and most eating establishments will provide containers for leftover portions [33]. Enhancing consumer educational efforts to reduce fruit and vegetable waste in the household will also be critically important. Consumers may benefit from increased knowledge about how to discern bruises/abrasions from spoilage, how to tell when fruits and vegetables are ripe, and how to prepare and safely store them [30, 34]. The Supplemental Nutrition Assistance Program Education, which provides education and guidance for low-income households to make healthy food choices [35], offers a key platform to increase consumer knowledge of strategies to reduce food waste.

This study is not without limitations. Data on food price inflation were available for only 15 major food categories (from CPI), whereas data on food intake were available for over 8500 individual foods (from NHANES), so linking these data may have contributed to over-generalized estimates of contemporary prices for individual foods. Lack of data availability also prevented analyses of food waste across divergent geographic regions and subpopulations, and more research is needed to estimate variability of food waste across these domains. Estimates of food waste were derived from the LAFA data series, which provides a single estimate of the proportion of each food wasted across years. This limitation prevented a time trend analysis, and required that the same food-specific waste proportions were applied consistently to FAH and FAFH. Furthermore, this limitation made it necessary to apply food-specific waste proportions consistently to all individuals in NHANES, which has precedent in previous studies [6, 36–38]. The need to develop innovative methods to merge these datasets, and the additional limitations of doing so, have been noted elsewhere [39]. Some of the datasets used in this study do not include uncertainty estimates (e.g., LAFA), which may have resulted in overly narrow confidence intervals. The expenditures presented in this study do not take into account the cost of household utilities (e.g., water, electricity, natural gas) and appliances used to prepare food, so the full cost of FAH may be greater. Finally, self-reported food intake is subject to measurement error from social desirability bias, with individuals likely over-reporting intake of perceived healthy foods and under-reporting intake of perceived unhealthy foods. Additionally, some individuals may have consumed a portion of their meal outside of the home but consumed the leftovers at home, which could have resulted in mis-categorization of FAH and FAFH in this study. Self-reported dietary data are nonetheless a rich source of information on intake of individual foods on a population level [40]. Overall, this study should not be interpreted as providing perfect estimates of food expenditure; instead, this study represents a novel approach to address important, interdisciplinary knowledge gaps using existing, publicly-available datasets.

The strengths of this study should be considered when interpreting the findings. For the first time, nationally-representative datasets on food intake, food waste, food prices, eating location, and food price inflation were linked to provide a robust measure of the daily per capita cost of consumer food wasted, inedible, and consumed in contemporary dollars. Importantly, this study also introduced a novel method of accounting for the important price differences between FAH and FAFH, thereby filling an important research gap [39]. The large sample size and multi-stage sampling structure of the source data make these findings generalizable to the US adult population, and the implications are far-ranging, from nutrition-related health outcomes to environmental sustainability.

## Conclusions

This study presents a fresh look at the daily per capita cost of consumer food waste, inedible portions, and consumed food in the US. We use an innovative approach that links dietary data collected from nearly 40 thousand adults over a 16-year period with nationally-representative data on food waste, food prices, eating location, and food price inflation. The average US adult spends over one-quarter of their food budget on food that ends up being wasted, more than the annual expenditure on vehicle gasoline, apparel, household heating and electricity, property taxes, and household maintenance and insurance. These results also shed new light on cost-effective ways to reduce food waste at the consumer level, by targeting waste reduction efforts on meat and seafood consumed outside of the home and fruits and vegetables consumed in the home. A number of strategies are available to help consumers reduce their food waste, which can increase their financial flexibility to purchase more healthy foods while simultaneously reducing environmental impact.

## Supplementary information

**Additional file 1.** Methodological uncertainty and assumptions embedded in data source linkages.

**Additional file 2 : Figure S1.** Steps to derive the proportion of food waste from the edible weight of food. Figure adapted, with permission, from Conrad, Zach; Niles, Meredith; Neher, Deb; Roy, Eric; Tichenor, Nicole; Jahns, Lisa. (2018). Relationship between diet quality, food waste, and environmental sustainability. *PLoS ONE,* 13:e0195405. Text boxes with solid outlines represent data aquired from USDA Loss-adjusted Food Availability data series (LAFA); text boxes with dashed outlines represent derived data.

**Additional file 3 : Table S1.** Foods included in analysis. NHANES, National Health and Nutrition Examination Survey; CPI, Consumer Price Index; FoodAPS, Food Acquisition and Purchase Survey. ^1^Approximately 80% of FoodAPS codes come from the USDA Food and Nutrient Database for Dietary Studes (FNDDS), and the remaining codes are manually assigned by FoodAPS staff (FoodAPS). ^2^Leading digits in each 8-digit food code. ^3^All codes begin with the prefix “CUUR0000S”. ^4^Leading digits in each 8- or 10-digit food code. ^5^Includes crackers and other grain-based snacks. ^6^Includes French toast and other sweet grain-based foods. ^7^Includes imitation milk, flavored milk and milk drinks, evaporated and condensed milk, and dry and powdered milk. ^8^Includes dairy-based sauces. ^9^Includes lamb, goat, and game. ^10^Includes sandwiches made from all meat and seafood sources, luncheon meats, and burgers. ^11^Includes butter and margarine. ^12^Includes water, alcohol, and beverage concentrates.

**Additional file 4 : Table S2.** Retail prices for foods purchased for consumption at home and away from home, 2012–2013 (*n* = 4305). Data acquired from the USDA National Household Food Acquisition and Purchase Survey, 2012–2013. ^1^Ratio of food away from home to food at home.

**Additional file 5 : Table S3.** Consumer Price Index and inflation coefficients, 2001–2016. NA, Not applicable. ^1^Represents 1 + percent change in Consumer Price Index from 2001 to 2016. ^2^Derived by (C_FAFH_/C_FAH_)C_i_, where *C* represents the inflation coefficient for all food away from home (*FAFH*), all food at home (*FAH*), and each individual food category (*i*).

**Additional file 6 : Table S4.** Daily per capita cost of food waste by food type, 2001–2016 (*n* = 39,758). 95% confidence intervals not listed for foods with waste <$0.01. ^1^Paired Wald test used to estimate the difference between food at home and food away from home. ^2^Includes crackers and other grain-based snacks. ^3^Includes French toast and other sweet grain-based foods. ^4^Includes imitation milk, flavored milk and milk drinks, evaporated and condensed milk, and dry and powdered milk. ^5^Includes dairy-based sauces. ^6^Includes lamb, goat, and game. ^7^Includes sandwiches made from all meat and seafood sources, luncheon meats, and burgers. ^8^Includes beverage concentrates. ^9^Includes butter and margarine. ^10^Includes water, alcohol, and beverage concentrates.

**Additional file 7 : Table S5.** Sensitivity analysis for food price assumptions, 2001–2016 (*n* = 39,758). Food purchased = wasted + inedible + consumed. Model 1: Expenditures estimated using 2008 dollar values for all foods reported consumed. Model 2: Expenditures estimated using food at home prices for all foods reported consumed. Model 3: Model 1 + Model 2. ^1^Difference between the estimated expenditure and each of the modeled expenditures tested using Wald tests at *P* < 0.05, with Bonferroni adjustment for multiple comparisons. Superscript letters indicate statistical significance for independent comparisons between estimated expenditure and Model 1 (^a^), Model 2 (^b^), and Model 3 (^c^).

**Additional file 8 : Table S6.** Sensitivity analysis for food intake assumptions, 2001–2016 (n = 39,758). Food purchased = wasted + inedible + consumed. Model 4: Expenditures estimated for individuals included in the National Health and Nutrition Examination Survey, 2001–2010 only. Model 5: Expenditures estimated for individuals included in the National Health and Nutrition Examination Survey, 2011–2016 only. ^1^Difference between the estimated expenditure and each of the modeled expenditures tested using Wald tests at *P* < 0.05, with Bonferroni adjustment for multiple comparisons. Superscript letters indicate statistical significance for independent comparisons between estimated expenditure and Model 4 (^a^) and Model 5 (^b^).

## Data Availability

The datasets used in this study are publicly available: https://wwwn.cdc.gov/nchs/nhanes/Default.aspx;http://fcid.foodrisk.org/#:https://www.ers.usda.gov/data-products/food-availability-per-capita-data-system/;https://www.ers.usda.gov/data-products/foodaps-national-household-food-acquisition-and-purchase-survey/

## Abbreviations

CNPP: Center for Nutrition Policy and Promotion
FAFH: Food away from home
FAH: Food at home
FCID: Food commodity intake database
FoodAPS: Food acquisition and purchase survey
NHANES: National Health and Nutrition Examination Survey
USDA: US Department of Agriculture
