# Supplementary material for: Daily cost of consumer food wasted, inedible, and consumed in the United States, 2001–2016
Source: Nutr J. 2020 Apr 20;19:35. doi: 10.1186/s12937-020-00552-w (PMC7168972; doi:10.1186/s12937-020-00552-w)
Supplement: Supplementary file 1 — Additional file 1. Methodological uncertainty and assumptions embedded in data source linkages. [file 12937_2020_552_MOESM1_ESM.docx]

Additional File 1: Methodological uncertainty and assumptions embedded in data source linkages

This file provides a detailed description of the methods used to link the numerous datasets used in this study, as well as embedded sources of uncertainty and assumptions. This process is depicted in Figure 1. The National Health and Nutrition Examination Survey (NHANES) forms the basis of this study, since it is a rich source of data on individual-level food intake and eating behaviors. However, NHANES does not provide data on food waste and food prices; although it does provide data on inedible portions of some foods, these data are not complete so we incorporate data on inedible portions from another source, which is discussed below. The methodology used in this study, and discussed here, was developed to extend the use of NHANES for purposes of estimating food waste and dietary expenditures. There are numerous sources of uncertainty in each of the data sources used in this methodology, and assumptions were necessarily made through the process of linking these data sources. Therefore, this methodology is not perfect, and we welcome other researchers to improve upon the methods described here.

**Estimating food wasted, inedible, and consumed**

This process links data on food intake from NHANES with data on food waste and inedible portions from the Loss-adjusted Food Availability data series (USDA).[1] To do this, the Food Commodity Intake Database (FCID) was used as an intermediary data source.[2] We have described this process previously[3], and we provide additional details below.

*Linking NHANES and FCID*

NHANES provides individual-level data on the amount of each food reported consumed. In some cases, individuals report eating distinct food items, like a tomato. Yet in most cases, individuals report consuming mixed dishes that contain multiple food items (i.e., ingredients), such as lasagna, which includes wheat, tomatoes, cheese, etc. NHANES does not provide data on the amount of each dish wasted and inedible (it does provide information on inedible portions for some individual foods, but not for most mixed dishes), so additional data sources were needed for this purpose.

The Food and Nutrient Database for Dietary Studies (FNDDS)[4] can be used to disaggregate NHANES dishes into their individual foods, but the degree of resolution is too course to be useful in the present study. For example, FNDDS will disaggregate lasagna into noodles and tomato sauce, but each of these contain multiple ingredients that are not disaggregated further, like wheat and tomatoes. This finer degree of resolution is needed to link with data on food waste and inedible portions from LAFA.[1] Thus, each NHANES mixed dish was disaggregated into its individual foods using FCID, which provides data on the weight of nearly 500 foods included in each NHANES dish.[2] This linkage has been pre-established by the US Environmental Protection Agency. The current iteration of FCID provides linkage to NHANES 2001-2010 only, and therefore does not account for new foods and reformulations that entered the market after that time period. Since we utilized NHANES data from 2001-2016, we conducted post hoc sensitivity analyses to examine the degree to which this biased our estimates of food expenditure (Supplemental Table 6). These sensitivity analyses demonstrated that, compared to 2001-2016 expenditures, 2001-2010 expenditures were $0.60 higher (P=0.034), and 2011-2016 expenditures were $0.91 lower (P<0.001). Therefore, our final estimation of food prices should be considered conservative.

*Linking FCID and LAFA*

LAFA provides estimates of waste and inedible portions for over 200 individual foods.[1] Each food in FCID was linked (hand-coded) with a distinct food in LAFA according to best matches of food descriptions. In our previous work,[3] two investigators matched these descriptions independently, and infrequent discrepancies were resolved through discussion and consensus. Successful matches were reached for 92% of the foods in FCID. The remainder of these foods were consumed infrequently and in insignificant amounts by the general population (such as passionfruit, arrowroot, and sea plants), and were not included in the analyses. All linkages are can be found in the table below.

| Dataset linkage for the Loss-adjusted Food Availability data series (LAFA) and Food Commodity Intake Database (FCID) | | | | | | |
| --- | --- | --- | --- | --- | --- | --- |
| LAFA description |  | FCID code |  | FCID description |  | Match^1^ |
| rye flour |  | 1500329000 |  | Rye, flour |  | 1 |
| canned apple and applesauce |  | 1100011001 |  | Apple, sauce-babyfood |  | 1 |
| pork |  | 3400290000 |  | Pork, meat |  | 1 |
| cane and beet sugar |  | 9500362001 |  | Sugarcane, sugar-babyfood |  | 1 |
| grape juice |  | 1304176001 |  | Grape, juice-babyfood |  | 1 |
| canned apple and applesauce |  | 1100011000 |  | Apple, sauce |  | 1 |
| corn starch |  | 1500123001 |  | Corn, field, starch-babyfood |  | 1 |
| dried plum |  | 1203286001 |  | Plum, prune, fresh-babyfood |  | 1 |
| apple juice |  | 1100010000 |  | Apple, juice |  | 1 |
| dried plum |  | 1203286000 |  | Plum, prune, fresh |  | 1 |
| mature pea and lentil |  | 603203000 |  | Lentil, seed |  | 1 |
| dry pear |  | 1100267000 |  | Pear, dried |  | 1 |
| turkey |  | 5000382001 |  | Turkey, meat-babyfood |  | 1 |
| black bean |  | 603030000 |  | Bean, black, seed |  | 1 |
| great northern bean |  | 603035000 |  | Bean, great northern, seed |  | 1 |
| dried onion |  | 301238000 |  | Onion, bulb, dried |  | 1 |
| prune juice |  | 1203288001 |  | Plum, prune, juice-babyfood |  | 1 |
| beef |  | 3100044000 |  | Beef, meat |  | 1 |
| honey |  | 9500186001 |  | Honey-babyfood |  | 1 |
| edible beef tallow |  | 3100047000 |  | Beef, fat |  | 1 |
| navy bean |  | 603040000 |  | Bean, navy, seed |  | 1 |
| dry peach |  | 1202261000 |  | Peach, dried |  | 1 |
| grapefruit juice |  | 1003181000 |  | Grapefruit, juice |  | 1 |
| egg |  | 7000147001 |  | Egg, yolk-babyfood |  | 1 |
| cranberry juice |  | 1307132001 |  | Cranberry, juice-babyfood |  | 1 |
| cranberry juice |  | 1307132000 |  | Cranberry, juice |  | 1 |
| dry fig |  | 2302154000 |  | Fig, dried |  | 1 |
| lemon juice |  | 1002200000 |  | Lemon, juice |  | 1 |
| potato chip |  | 103296000 |  | Potato, chips |  | 1 |
| egg |  | 7000146000 |  | Egg, white |  | 1 |
| chicken |  | 4000093001 |  | Chicken, meat-babyfood |  | 1 |
| egg |  | 7000147000 |  | Egg, yolk |  | 1 |
| macadamia nut |  | 1400213000 |  | Macadamia nut |  | 1 |
| corn flour and meal |  | 1500120000 |  | Corn, field, flour |  | 1 |
| orange juice |  | 1001241000 |  | Orange, juice |  | 1 |
| rye flour |  | 1500328000 |  | Rye, grain |  | 1 |
| dried plum |  | 1203287000 |  | Plum, prune, dried |  | 1 |
| salad and cooking oil |  | 1500125000 |  | Corn, field, oil |  | 1 |
| honey |  | 9500186000 |  | Honey |  | 1 |
| lemon juice |  | 1002200001 |  | Lemon, juice-babyfood |  | 1 |
| white, whole wheat, and durum flour |  | 1500401000 |  | Wheat, grain |  | 1 |
| high fructose corn sweetener |  | 1500124000 |  | Corn, field, syrup |  | 1 |
| beef |  | 3100044001 |  | Beef, meat-babyfood |  | 1 |
| pineapple juice |  | 2403281001 |  | Pineapple, juice-babyfood |  | 1 |
| grape juice |  | 1304176000 |  | Grape, juice |  | 1 |
| dried potato |  | 103298001 |  | Potato, flour-babyfood |  | 1 |
| dried potato |  | 103298000 |  | Potato, flour |  | 1 |
| lime juice |  | 1002207000 |  | Lime, juice |  | 1 |
| cane and beet sugar |  | 101052000 |  | Beet, sugar |  | 1 |
| hazelnut |  | 1400155000 |  | Hazelnut |  | 1 |
| peanut |  | 9500263000 |  | Peanut |  | 1 |
| apple juice |  | 1100010001 |  | Apple, juice-babyfood |  | 1 |
| cane and beet sugar |  | 101052001 |  | Beet, sugar-babyfood |  | 1 |
| white, whole wheat, and durum flour |  | 1500401001 |  | Wheat, grain-babyfood |  | 1 |
| corn flour and meal |  | 1500120001 |  | Corn, field, flour-babyfood |  | 1 |
| white, whole wheat, and durum flour |  | 1500402001 |  | Wheat, flour-babyfood |  | 1 |
| corn starch |  | 1500123000 |  | Corn, field, starch |  | 1 |
| dried potato |  | 103297001 |  | Potato, dry (granules/ flakes)-babyfood |  | 1 |
| pork |  | 3400290001 |  | Pork, meat-babyfood |  | 1 |
| almond |  | 1400003000 |  | Almond |  | 1 |
| white, whole wheat, and durum flour |  | 1500404000 |  | Wheat, bran |  | 1 |
| dried potato |  | 103297000 |  | Potato, dry (granules/ flakes) |  | 1 |
| turkey |  | 5000382000 |  | Turkey, meat |  | 1 |
| mature lima bean |  | 603038000 |  | Bean, lima, seed |  | 1 |
| raisin |  | 1304178000 |  | Grape, raisin |  | 1 |
| lard |  | 3400293001 |  | Pork, fat-babyfood |  | 1 |
| dried date |  | 2303141000 |  | Date |  | 1 |
| orange juice |  | 1001241001 |  | Orange, juice-babyfood |  | 1 |
| cane and beet sugar |  | 9500362000 |  | Sugarcane, sugar |  | 1 |
| edible syrup |  | 1500345000 |  | Sorghum, syrup |  | 1 |
| lard |  | 3400293000 |  | Pork, fat |  | 1 |
| dried apple |  | 1100009000 |  | Apple, dried |  | 1 |
| egg |  | 7000145000 |  | Egg, whole |  | 1 |
| prune juice |  | 1203288000 |  | Plum, prune, juice |  | 1 |
| dried apricot |  | 1202013000 |  | Apricot, dried |  | 1 |
| white, whole wheat, and durum flour |  | 1500402000 |  | Wheat, flour |  | 1 |
| pinto bean |  | 603042000 |  | Bean, pinto, seed |  | 1 |
| edible beef tallow |  | 3100047001 |  | Beef, fat-babyfood |  | 1 |
| chicken |  | 4000093000 |  | Chicken, meat |  | 1 |
| pineapple juice |  | 2403281000 |  | Pineapple, juice |  | 1 |
| pistachio |  | 1400282000 |  | Pistachio |  | 1 |
| dried onion |  | 301238001 |  | Onion, bulb, dried-babyfood |  | 1 |
| peanut |  | 9500264000 |  | Peanut, butter |  | 1 |
| high fructose corn sweetener |  | 1500124001 |  | Corn, field, syrup-babyfood |  | 1 |
| red kidney bean |  | 603036000 |  | Bean, kidney, seed |  | 1 |
| pecan |  | 1400269000 |  | Pecan |  | 1 |
| walnut |  | 1400391000 |  | Walnut |  | 1 |
| white, whole wheat, and durum flour |  | 1500403000 |  | Wheat, germ |  | 1 |
| lime juice |  | 1002207001 |  | Lime, juice-babyfood |  | 1 |
| dried apple |  | 1100009001 |  | Apple, dried-babyfood |  | 1 |
| corn flour and meal |  | 1500121000 |  | Corn, field, meal |  | 1 |
| egg |  | 7000145001 |  | Egg, whole-babyfood |  | 1 |
| barley |  | 1500025001 |  | Barley, pearled barley-babyfood |  | 2 |
| other mature bean |  | 602031000 |  | Bean, broad, succulent |  | 2 |
| fresh banana |  | 2402023001 |  | Banana-babyfood |  | 2 |
| fresh mango |  | 2402215001 |  | Mango-babyfood |  | 2 |
| chicken |  | 4000097000 |  | Chicken, skin |  | 2 |
| oats |  | 1500233001 |  | Oat, groats/rolled oats-babyfood |  | 2 |
| rice |  | 1500326001 |  | Rice, bran-babyfood |  | 2 |
| fresh strawberry |  | 1307359001 |  | Strawberry-babyfood |  | 2 |
| oats |  | 1500232001 |  | Oat, flour-babyfood |  | 2 |
| lamb |  | 3500343000 |  | Sheep, liver |  | 2 |
| rice |  | 1500405000 |  | Wild rice |  | 2 |
| fresh blueberry |  | 1302057000 |  | Blueberry |  | 2 |
| fresh endive |  | 401150000 |  | Endive |  | 2 |
| other mature bean |  | 603098001 |  | Chickpea, seed-babyfood |  | 2 |
| fresh lemon |  | 1002199000 |  | Lemon |  | 2 |
| cane and beet sugar |  | 101053000 |  | Beet, sugar, molasses |  | 2 |
| fresh apple |  | 1100008001 |  | Apple, peeled fruit-babyfood |  | 2 |
| chicken |  | 6000304000 |  | Poultry, other, fat |  | 2 |
| fresh squash |  | 902356000 |  | Squash, summer |  | 2 |
| other oil |  | 2003128001 |  | Cottonseed, oil-babyfood |  | 2 |
| beef |  | 3100046001 |  | Beef, meat byproducts-babyfood |  | 2 |
| fresh kale |  | 402194000 |  | Kale |  | 2 |
| rice |  | 1500323000 |  | Rice, white |  | 2 |
| fresh onion |  | 301237001 |  | Onion, bulb-babyfood |  | 2 |
| mature pea and lentil |  | 603256001 |  | Pea, dry-babyfood |  | 2 |
| turkey |  | 5000384001 |  | Turkey, meat byproducts-babyfood |  | 2 |
| other mature bean |  | 603032000 |  | Bean, broad, seed |  | 2 |
| turkey |  | 5000385000 |  | Turkey, fat |  | 2 |
| other mature bean |  | 603039000 |  | Bean, mung, seed |  | 2 |
| fresh pineapple |  | 2403280000 |  | Pineapple, dried |  | 2 |
| mature pea and lentil |  | 603256000 |  | Pea, dry |  | 2 |
| lamb |  | 3500341000 |  | Sheep, fat |  | 2 |
| fresh okra |  | 802234000 |  | Okra |  | 2 |
| fresh snap bean |  | 601043000 |  | Bean, snap, succulent |  | 2 |
| fresh squash |  | 902357001 |  | Squash, winter-babyfood |  | 2 |
| other mature bean |  | 600347000 |  | Soybean, seed |  | 2 |
| frozen blackberry |  | 1301055000 |  | Blackberry |  | 2 |
| coconut |  | 1400112000 |  | Coconut, dried |  | 2 |
| fresh potato |  | 103299000 |  | Potato, tuber, w/peel |  | 2 |
| barley |  | 1500026001 |  | Barley, flour-babyfood |  | 2 |
| other tree nut |  | 1400068000 |  | Butternut |  | 2 |
| other oil |  | 2002365000 |  | Sunflower, oil |  | 2 |
| lamb |  | 3500342000 |  | Sheep, kidney |  | 2 |
| fresh bell pepper |  | 802270000 |  | Pepper, bell |  | 2 |
| turkey |  | 5000385001 |  | Turkey, fat-babyfood |  | 2 |
| fresh tomato |  | 801375000 |  | Tomato |  | 2 |
| other oil |  | 2003128000 |  | Cottonseed, oil |  | 2 |
| fresh celery |  | 2202085000 |  | Celery |  | 2 |
| lamb |  | 3500341001 |  | Sheep, fat-babyfood |  | 2 |
| fresh garlic |  | 301165000 |  | Garlic, bulb |  | 2 |
| beef |  | 3100045000 |  | Beef, meat, dried |  | 2 |
| fresh apple |  | 1100007000 |  | Apple, fruit with peel |  | 2 |
| fresh grape |  | 1304175000 |  | Grape |  | 2 |
| fresh artichoke |  | 9500016000 |  | Artichoke, globe |  | 2 |
| canned tuna |  | 8000159000 |  | Fish-saltwater finfish, tuna |  | 2 |
| other mature bean |  | 9500109000 |  | Cocoa bean, chocolate |  | 2 |
| fresh banana |  | 2402024001 |  | Banana, dried-babyfood |  | 2 |
| fresh tomato |  | 801375001 |  | Tomato-babyfood |  | 2 |
| other oil |  | 2002330000 |  | Safflower, oil |  | 2 |
| chicken |  | 4000097001 |  | Chicken, skin-babyfood |  | 2 |
| fresh cherry |  | 1201090000 |  | Cherry |  | 2 |
| other oil |  | 2001319000 |  | Rapeseed, oil |  | 2 |
| fresh cranberry |  | 1307130000 |  | Cranberry |  | 2 |
| corn flour and meal |  | 1500122000 |  | Corn, field, bran |  | 2 |
| other oil |  | 2002365001 |  | Sunflower, oil-babyfood |  | 2 |
| fresh cranberry |  | 1307130001 |  | Cranberry-babyfood |  | 2 |
| fresh plum |  | 1203285000 |  | Plum |  | 2 |
| oats |  | 1500232000 |  | Oat, flour |  | 2 |
| fresh cauliflower |  | 500083000 |  | Cauliflower |  | 2 |
| rice |  | 1500324000 |  | Rice, brown |  | 2 |
| fresh watermelon |  | 901399000 |  | Watermelon |  | 2 |
| other tree nut |  | 1400092000 |  | Chestnut |  | 2 |
| canned tomato |  | 801377000 |  | Tomato, puree |  | 2 |
| fresh orange |  | 1001242000 |  | Orange, peel |  | 2 |
| fresh papaya |  | 2402245000 |  | Papaya |  | 2 |
| lamb |  | 3500339001 |  | Sheep, meat-babyfood |  | 2 |
| fresh apricot |  | 1202012000 |  | Apricot |  | 2 |
| fresh pear |  | 1100266001 |  | Pear-babyfood |  | 2 |
| turkey |  | 5000386001 |  | Turkey, skin-babyfood |  | 2 |
| fresh pineapple |  | 2403279000 |  | Pineapple |  | 2 |
| beef |  | 3100049000 |  | Beef, liver |  | 2 |
| fresh plum |  | 1203285001 |  | Plum-babyfood |  | 2 |
| fresh lemon |  | 1002201000 |  | Lemon, peel |  | 2 |
| fresh raspberry |  | 1301320001 |  | Raspberry-babyfood |  | 2 |
| other tree nut |  | 1400081000 |  | Cashew |  | 2 |
| fresh and frozen fish |  | 8000157000 |  | Fish-freshwater finfish |  | 2 |
| fresh leaf lettuce |  | 401205000 |  | Lettuce, leaf |  | 2 |
| fresh bell pepper |  | 802271000 |  | Pepper, bell, dried |  | 2 |
| fresh broccoli |  | 500061001 |  | Broccoli-babyfood |  | 2 |
| fresh pineapple |  | 2403279001 |  | Pineapple-babyfood |  | 2 |
| other oil |  | 9500244001 |  | Palm, oil-babyfood |  | 2 |
| fresh cherry |  | 1201090001 |  | Cherry-babyfood |  | 2 |
| fresh mustard green |  | 402229000 |  | Mustard greens |  | 2 |
| chicken |  | 6000305000 |  | Poultry, other, skin |  | 2 |
| fresh collard green |  | 402117000 |  | Collards |  | 2 |
| fresh sweet corn |  | 1500127000 |  | Corn, sweet |  | 2 |
| chicken |  | 6000301000 |  | Poultry, other, meat |  | 2 |
| other oil |  | 1400114000 |  | Coconut, oil |  | 2 |
| other oil |  | 1400114001 |  | Coconut, oil-babyfood |  | 2 |
| fresh celery |  | 2202085001 |  | Celery-babyfood |  | 2 |
| fresh pumpkin |  | 902308000 |  | Pumpkin |  | 2 |
| frozen green pea |  | 602255000 |  | Pea, succulent |  | 2 |
| salad and cooking oil |  | 2301236000 |  | Olive, oil |  | 2 |
| fresh apricot |  | 1202012001 |  | Apricot-babyfood |  | 2 |
| fresh snap bean |  | 601043001 |  | Bean, snap, succulent-babyfood |  | 2 |
| oats |  | 1500233000 |  | Oat, groats/rolled oats |  | 2 |
| barley |  | 1500027000 |  | Barley, bran |  | 2 |
| fresh sweet potato |  | 103407000 |  | Yam bean |  | 2 |
| fresh and frozen shellfish |  | 8000162000 |  | Fish-shellfish, mollusc |  | 2 |
| other mature bean |  | 603041000 |  | Bean, pink, seed |  | 2 |
| fresh carrot |  | 101078001 |  | Carrot-babyfood |  | 2 |
| other oil |  | 9500244000 |  | Palm, oil |  | 2 |
| other oil |  | 2002330001 |  | Safflower, oil-babyfood |  | 2 |
| fresh tomato |  | 801378001 |  | Tomato, dried-babyfood |  | 2 |
| other mature bean |  | 600349000 |  | Soybean, soy milk |  | 2 |
| fresh grapefruit |  | 1003180000 |  | Grapefruit |  | 2 |
| canned tomato |  | 801377001 |  | Tomato, puree-babyfood |  | 2 |
| fresh peach |  | 1202260001 |  | Peach-babyfood |  | 2 |
| fresh sweet potato |  | 103406000 |  | Yam, true |  | 2 |
| fresh blueberry |  | 1302057001 |  | Blueberry-babyfood |  | 2 |
| turkey |  | 5000384000 |  | Turkey, meat byproducts |  | 2 |
| salad and cooking oil |  | 9500265000 |  | Peanut, oil |  | 2 |
| salad and cooking oil |  | 1500125001 |  | Corn, field, oil-babyfood |  | 2 |
| fresh mango |  | 2402216000 |  | Mango, dried |  | 2 |
| other oil |  | 1400004000 |  | Almond, oil |  | 2 |
| fresh and frozen fish |  | 8000158000 |  | Fish-freshwater finfish, farm raised |  | 2 |
| barley |  | 1500026000 |  | Barley, flour |  | 2 |
| rice |  | 1500325001 |  | Rice, flour-babyfood |  | 2 |
| fresh pumpkin |  | 902309000 |  | Pumpkin, seed |  | 2 |
| fresh sweet potato |  | 103366000 |  | Sweet potato |  | 2 |
| fresh honeydew |  | 901187000 |  | Honeydew melon |  | 2 |
| other oil |  | 2001337000 |  | Sesame, oil |  | 2 |
| other mature bean |  | 603182000 |  | Guar, seed |  | 2 |
| frozen green pea |  | 601257000 |  | Pea, edible podded, succulent |  | 2 |
| fresh kiwi |  | 1304195000 |  | Kiwifruit, fuzzy |  | 2 |
| other tree nut |  | 1400059000 |  | Brazil nut |  | 2 |
| other mature bean |  | 9500110000 |  | Cocoa bean, powder |  | 2 |
| fresh papaya |  | 2402246000 |  | Papaya, dried |  | 2 |
| fresh apple |  | 1100008000 |  | Apple, peeled fruit |  | 2 |
| canned tomato |  | 801376000 |  | Tomato, paste |  | 2 |
| lamb |  | 3500340000 |  | Sheep, meat byproducts |  | 2 |
| fresh carrot |  | 101079000 |  | Carrot, juice |  | 2 |
| fresh squash |  | 902356001 |  | Squash, summer-babyfood |  | 2 |
| other oil |  | 2001163000 |  | Flax seed, oil |  | 2 |
| salad and cooking oil |  | 600350000 |  | Soybean, oil |  | 2 |
| fresh strawberry |  | 1307359000 |  | Strawberry |  | 2 |
| salad and cooking oil |  | 600350001 |  | Soybean, oil-babyfood |  | 2 |
| edible syrup |  | 9500219000 |  | Maple syrup |  | 2 |
| fresh bell pepper |  | 802271001 |  | Pepper, bell, dried-babyfood |  | 2 |
| chicken |  | 4000095001 |  | Chicken, meat byproducts-babyfood |  | 2 |
| fresh cantaloupe |  | 901075000 |  | Cantaloupe |  | 2 |
| other oil |  | 9500276000 |  | Peppermint, oil |  | 2 |
| canned olive |  | 2301235000 |  | Olive |  | 2 |
| fresh eggplant |  | 802148000 |  | Eggplant |  | 2 |
| fresh squash |  | 902357000 |  | Squash, winter |  | 2 |
| rice |  | 1500325000 |  | Rice, flour |  | 2 |
| fresh potato |  | 103300000 |  | Potato, tuber, w/o peel |  | 2 |
| cane and beet sugar |  | 9500363000 |  | Sugarcane, molasses |  | 2 |
| fresh broccoli |  | 500061000 |  | Broccoli |  | 2 |
| fresh pear |  | 1100266000 |  | Pear |  | 2 |
| coconut |  | 1400111001 |  | Coconut, meat-babyfood |  | 2 |
| oats |  | 1500231000 |  | Oat, bran |  | 2 |
| fresh spinach |  | 401355000 |  | Spinach |  | 2 |
| fresh asparagus |  | 2201019000 |  | Asparagus |  | 2 |
| chicken |  | 6000303000 |  | Poultry, other, meat byproducts |  | 2 |
| fresh mushroom |  | 2100228000 |  | Mushroom |  | 2 |
| turkey |  | 5000386000 |  | Turkey, skin |  | 2 |
| fresh and frozen shellfish |  | 8000161000 |  | Fish-shellfish, crustacean |  | 2 |
| other oil |  | 2001319001 |  | Rapeseed, oil-babyfood |  | 2 |
| turkey |  | 5000383000 |  | Turkey, liver |  | 2 |
| mature lima bean |  | 602037000 |  | Bean, lima, succulent |  | 2 |
| pork |  | 3400292000 |  | Pork, meat byproducts |  | 2 |
| pork |  | 3400295000 |  | Pork, liver |  | 2 |
| rice |  | 1500323001 |  | Rice, white-babyfood |  | 2 |
| pork |  | 3400294000 |  | Pork, kidney |  | 2 |
| fresh potato |  | 103300001 |  | Potato, tuber, w/o peel-babyfood |  | 2 |
| fresh tangerine |  | 1001369000 |  | Tangerine |  | 2 |
| fresh cucumber |  | 902135000 |  | Cucumber |  | 2 |
| fresh tomato |  | 801378000 |  | Tomato, dried |  | 2 |
| canned tomato |  | 801376001 |  | Tomato, paste-babyfood |  | 2 |
| chicken |  | 4000096000 |  | Chicken, fat |  | 2 |
| coconut |  | 1400113000 |  | Coconut, milk |  | 2 |
| fresh raspberry |  | 1301320000 |  | Raspberry |  | 2 |
| other mature bean |  | 600348000 |  | Soybean, flour |  | 2 |
| other mature bean |  | 600348001 |  | Soybean, flour-babyfood |  | 2 |
| fresh orange |  | 1001240000 |  | Orange |  | 2 |
| fresh garlic |  | 301165001 |  | Garlic, bulb-babyfood |  | 2 |
| dry fig |  | 2302153000 |  | Fig |  | 2 |
| fresh radish |  | 101314000 |  | Radish, roots |  | 2 |
| fresh sweet corn |  | 1500127001 |  | Corn, sweet-babyfood |  | 2 |
| coconut |  | 1400111000 |  | Coconut, meat |  | 2 |
| chicken |  | 4000094000 |  | Chicken, liver |  | 2 |
| fresh brussels sprout |  | 500064000 |  | Brussels sprouts |  | 2 |
| lamb |  | 3500339000 |  | Sheep, meat |  | 2 |
| cane and beet sugar |  | 101053001 |  | Beet, sugar, molasses-babyfood |  | 2 |
| fresh and frozen fish |  | 8000160000 |  | Fish-saltwater finfish, other |  | 2 |
| other mature bean |  | 603098000 |  | Chickpea, seed |  | 2 |
| chicken |  | 6000302000 |  | Poultry, other, liver |  | 2 |
| barley |  | 1500025000 |  | Barley, pearled barley |  | 2 |
| beef |  | 3100046000 |  | Beef, meat byproducts |  | 2 |
| fresh peach |  | 1202260000 |  | Peach |  | 2 |
| mature pea and lentil |  | 602259000 |  | Pea, pigeon, succulent |  | 2 |
| mature pea and lentil |  | 603258000 |  | Pea, pigeon, seed |  | 2 |
| other mature bean |  | 603034000 |  | Bean, cowpea, seed |  | 2 |
| fresh lime |  | 1002206000 |  | Lime |  | 2 |
| rice |  | 1500326000 |  | Rice, bran |  | 2 |
| pork |  | 3400291000 |  | Pork, skin |  | 2 |
| frozen green pea |  | 602255001 |  | Pea, succulent-babyfood |  | 2 |
| fresh head lettuce |  | 401204000 |  | Lettuce, head |  | 2 |
| pork |  | 3400292001 |  | Pork, meat byproducts-babyfood |  | 2 |
| chicken |  | 4000095000 |  | Chicken, meat byproducts |  | 2 |
| fresh avocado |  | 2402020000 |  | Avocado |  | 2 |
| fresh mango |  | 2402215000 |  | Mango |  | 2 |
| fresh carrot |  | 101078000 |  | Carrot |  | 2 |
| beef |  | 3100048000 |  | Beef, kidney |  | 2 |
| other mature bean |  | 602033000 |  | Bean, cowpea, succulent |  | 2 |
| chicken |  | 4000096001 |  | Chicken, fat-babyfood |  | 2 |
| fresh sweet potato |  | 103366001 |  | Sweet potato-babyfood |  | 2 |
| fresh banana |  | 2402023000 |  | Banana |  | 2 |
| rice |  | 1500324001 |  | Rice, brown-babyfood |  | 2 |
| other tree nut |  | 1400278000 |  | Pine nut |  | 2 |
| fresh banana |  | 2402024000 |  | Banana, dried |  | 2 |
| fresh turnip green |  | 402389000 |  | Turnip, greens |  | 2 |
| cane and beet sugar |  | 9500363001 |  | Sugarcane, molasses-babyfood |  | 2 |
| fresh spinach |  | 401355001 |  | Spinach-babyfood |  | 2 |
| fresh cabbage |  | 500069000 |  | Cabbage |  | 2 |
| fresh onion |  | 301237000 |  | Onion, bulb |  | 2 |
| beef |  | 3100049001 |  | Beef, liver-babyfood |  | 2 |
| fresh radish |  | 103082001 |  | Cassava-babyfood |  | 3 |
| fresh cabbage |  | 500071000 |  | Cabbage, Chinese, napa |  | 3 |
| fresh collard green |  | 9500177000 |  | Grape, leaves |  | 3 |
| fresh radish |  | 101050001 |  | Beet, garden, roots-babyfood |  | 3 |
| fresh apricot |  | 1202014000 |  | Apricot, juice |  | 3 |
| fresh strawberry |  | 1307360000 |  | Strawberry, juice |  | 3 |
| fresh turnip green |  | 1901334000 |  | Savory |  | 3 |
| nonfat dry milk |  | 3600223001 |  | Milk, nonfat solids-baby food/infant formula | | 3 |
| fresh turnip green |  | 9500188000 |  | Hop |  | 3 |
| barley |  | 2001162900 |  | Flax, seed |  | 3 |
| fresh pear |  | 1100268001 |  | Pear, juice-babyfood |  | 3 |
| fresh turnip green |  | 1901249000 |  | Parsley, dried leaves |  | 3 |
| fresh turnip green |  | 1901184001 |  | Herbs, other-babyfood |  | 3 |
| fresh turnip green |  | 401005000 |  | Amaranth, leafy |  | 3 |
| fresh pear |  | 1100310000 |  | Quince |  | 3 |
| fresh papaya |  | 2402247000 |  | Papaya, juice |  | 3 |
| fresh radish |  | 101316000 |  | Radish, Oriental, roots |  | 3 |
| fresh radish |  | 101084000 |  | Celeriac |  | 3 |
| fresh turnip green |  | 401138000 |  | Dandelion, leaves |  | 3 |
| grape juice |  | 1304179000 |  | Grape, wine and sherry |  | 3 |
| fresh cranberry |  | 1302174000 |  | Gooseberry |  | 3 |
| white, whole wheat, and durum flour |  | 1500226000 |  | Millet, grain |  | 3 |
| fresh banana |  | 2402284000 |  | Plantain, dried |  | 3 |
| fresh plum |  | 2402277000 |  | Persimmon |  | 3 |
| fresh radish |  | 103166000 |  | Ginger |  | 3 |
| fresh tomato |  | 801374000 |  | Tomatillo |  | 3 |
| fresh pear |  | 902021000 |  | Balsam pear |  | 3 |
| fresh apple |  | 2403346000 |  | Soursop |  | 3 |
| heavy cream |  | 3600222000 |  | Milk, fat |  | 3 |
| fresh collard green |  | 2201243000 |  | Palm heart, leaves |  | 3 |
| fresh radish |  | 101251001 |  | Parsnip-babyfood |  | 3 |
| fresh celery |  | 2202322000 |  | Rhubarb |  | 3 |
| mature pea and lentil |  | 2302077000 |  | Carob |  | 3 |
| fresh turnip green |  | 1901184000 |  | Herbs, other |  | 3 |
| fresh cranberry |  | 2401211000 |  | Lychee |  | 3 |
| fresh collard green |  | 401367000 |  | Swiss chard |  | 3 |
| fresh lemon |  | 1001106000 |  | Citron |  | 3 |
| fresh leaf lettuce |  | 402018000 |  | Arugula |  | 3 |
| heavy cream |  | 3600222001 |  | Milk, fat-baby food/infant formula |  | 3 |
| fresh turnip green |  | 401118000 |  | Cilantro, leaves |  | 3 |
| barley |  | 1500065000 |  | Buckwheat |  | 3 |
| fresh onion |  | 301338000 |  | Shallot, bulb |  | 3 |
| fresh mango |  | 2402217000 |  | Mango, juice |  | 3 |
| fresh mango |  | 2402217001 |  | Mango, juice-babyfood |  | 3 |
| fresh radish |  | 101100000 |  | Chicory, roots |  | 3 |
| fresh radish |  | 101168000 |  | Ginseng, dried |  | 3 |
| fresh turnip green |  | 1901220001 |  | Marjoram-babyfood |  | 3 |
| fresh turnip green |  | 402398000 |  | Watercress |  | 3 |
| fresh radish |  | 101067000 |  | Burdock |  | 3 |
| fresh squash |  | 902102000 |  | Chinese waxgourd |  | 3 |
| fresh apricot |  | 1202014001 |  | Apricot, juice-babyfood |  | 3 |
| fresh radish |  | 101327000 |  | Rutabaga |  | 3 |
| fresh radish |  | 101388000 |  | Turnip, roots |  | 3 |
| fresh leaf lettuce |  | 200140000 |  | Dasheen, leaves |  | 3 |
| fresh cranberry |  | 1302137000 |  | Currant, dried |  | 3 |
| fresh cranberry |  | 1302136000 |  | Currant |  | 3 |
| fresh turnip green |  | 200101000 |  | Chicory, tops |  | 3 |
| fresh radish |  | 2201196000 |  | Kohlrabi |  | 3 |
| fresh watermelon |  | 901400000 |  | Watermelon, juice |  | 3 |
| fresh raspberry |  | 1301321001 |  | Raspberry, juice-babyfood |  | 3 |
| fresh papaya |  | 2302183000 |  | Guava |  | 3 |
| fresh radish |  | 101050000 |  | Beet, garden, roots |  | 3 |
| fresh tangerine |  | 1001370000 |  | Tangerine, juice |  | 3 |
| fresh turnip green |  | 1901028000 |  | Basil, fresh leaves |  | 3 |
| frozen blackberry |  | 1301058000 |  | Boysenberry |  | 3 |
| fresh strawberry |  | 1307360001 |  | Strawberry, juice-babyfood |  | 3 |
| fresh onion |  | 302239000 |  | Onion, green |  | 3 |
| fresh collard green |  | 103371000 |  | Tanier, corm |  | 3 |
| fresh turnip green |  | 1901029001 |  | Basil, dried leaves-babyfood |  | 3 |
| fresh onion |  | 302103000 |  | Chive, fresh leaves |  | 3 |
| fresh orange |  | 1002197000 |  | Kumquat |  | 3 |
| fresh celery |  | 2202086000 |  | Celery, juice |  | 3 |
| fresh cherry |  | 1201091000 |  | Cherry, juice |  | 3 |
| fresh tomato |  | 801379000 |  | Tomato, juice |  | 3 |
| fresh turnip green |  | 1901249001 |  | Parsley, dried leaves-babyfood |  | 3 |
| fresh turnip green |  | 401144000 |  | Dillweed |  | 3 |
| fresh radish |  | 103167000 |  | Ginger, dried |  | 3 |
| fresh turnip green |  | 200051000 |  | Beet, garden, tops |  | 3 |
| fresh radish |  | 103139000 |  | Dasheen, corm |  | 3 |
| beef |  | 3900312000 |  | Rabbit, meat |  | 3 |
| fresh turnip green |  | 401248000 |  | Parsley, leaves |  | 3 |
| fresh turnip green |  | 402133000 |  | Cress, garden |  | 3 |
| dry fig |  | 2403060000 |  | Breadfruit |  | 3 |
| fresh broccoli |  | 402062000 |  | Broccoli, Chinese |  | 3 |
| fresh radish |  | 103017000 |  | Artichoke, Jerusalem |  | 3 |
| white, whole wheat, and durum flour |  | 1500381000 |  | Triticale, flour |  | 3 |
| fresh peach |  | 1202262001 |  | Peach, juice-babyfood |  | 3 |
| fresh banana |  | 2402283000 |  | Plantain |  | 3 |
| fresh cranberry |  | 1307131000 |  | Cranberry, dried |  | 3 |
| fresh cabbage |  | 401313000 |  | Radicchio |  | 3 |
| fresh sweet corn |  | 1500126000 |  | Corn, pop |  | 3 |
| edible beef tallow |  | 3200171000 |  | Goat, fat |  | 3 |
| beef |  | 3800221000 |  | Meat, game |  | 3 |
| frozen blackberry |  | 1301056000 |  | Blackberry, juice |  | 3 |
| fresh turnip green |  | 1901029000 |  | Basil, dried leaves |  | 3 |
| fresh pear |  | 1100268000 |  | Pear, juice |  | 3 |
| fresh radish |  | 101190000 |  | Horseradish |  | 3 |
| fresh radish |  | 103387000 |  | Turmeric |  | 3 |
| fresh turnip green |  | 401104000 |  | Chrysanthemum, garland |  | 3 |
| fresh strawberry |  | 2302358000 |  | Starfruit |  | 3 |
| fresh cherry |  | 1201091001 |  | Cherry, juice-babyfood |  | 3 |
| white, whole wheat, and durum flour |  | 9500006000 |  | Amaranth, grain |  | 3 |
| fresh radish |  | 101251000 |  | Parsnip |  | 3 |
| fresh peach |  | 1202262000 |  | Peach, juice |  | 3 |
| fresh peach |  | 1202230000 |  | Nectarine |  | 3 |
| frozen blackberry |  | 1301056001 |  | Blackberry, juice-babyfood |  | 3 |
| fresh onion |  | 302198000 |  | Leek |  | 3 |
| beef |  | 3200169000 |  | Goat, meat |  | 3 |
| nonfat dry milk |  | 3600223000 |  | Milk, nonfat solids |  | 3 |
| mature pea and lentil |  | 2302368000 |  | Tamarind |  | 3 |
| fresh endive |  | 9500054000 |  | Belgium endive |  | 3 |
| skim milk |  | 3600224000 |  | Milk, water |  | 3 |
| fresh onion |  | 2201152000 |  | Fennel, Florence |  | 3 |
| skim milk |  | 3600224001 |  | Milk, water-babyfood/infant formula |  | 3 |
| fresh cabbage |  | 500072000 |  | Cabbage, Chinese, mustard |  | 3 |
| fresh radish |  | 103082000 |  | Cassava |  | 3 |
| fresh lime |  | 2401351000 |  | Spanish lime |  | 3 |
| beef |  | 3200170000 |  | Goat, meat byproducts |  | 3 |
| fresh turnip green |  | 1901220000 |  | Marjoram |  | 3 |
| fresh raspberry |  | 1301321000 |  | Raspberry, juice |  | 3 |
| fresh lemon |  | 1001107000 |  | Citrus hybrids |  | 3 |
| fresh cabbage |  | 402070000 |  | Cabbage, Chinese, bok choy |  | 3 |
| fresh apple |  | 2403361000 |  | Sugar apple |  | 3 |
| frozen blackberry |  | 1303227000 |  | Mulberry |  | 3 |
| fresh cranberry |  | 1302191000 |  | Huckleberry |  | 3 |
| NA |  | 1902119000 |  | Coriander, seed |  | 4 |
| NA |  | 9500335001 |  | Seaweed-babyfood |  | 4 |
| NA |  | 2402290000 |  | Pomegranate, juice |  | 4 |
| NA |  | 1902105000 |  | Cinnamon |  | 4 |
| NA |  | 9500373000 |  | Tea, instant |  | 4 |
| NA |  | 802273000 |  | Pepper, nonbell, dried |  | 4 |
| NA |  | 2002364000 |  | Sunflower, seed |  | 4 |
| NA |  | 1800002000 |  | Alfalfa, seed |  | 4 |
| NA |  | 1902143000 |  | Dill, seed |  | 4 |
| NA |  | 103015000 |  | Arrowroot, flour |  | 4 |
| NA |  | 9500397000 |  | Water chestnut |  | 4 |
| NA |  | 902088000 |  | Chayote, fruit |  | 4 |
| NA |  | 1902354000 |  | Spices, other |  | 4 |
| NA |  | 1902354001 |  | Spices, other-babyfood |  | 4 |
| NA |  | 9500115000 |  | Coffee, roasted bean |  | 4 |
| NA |  | 2405252000 |  | Passionfruit |  | 4 |
| NA |  | 3600225001 |  | Milk, sugar (lactose)-baby food/infant formula | | 4 |
| NA |  | 2402289000 |  | Pomegranate |  | 4 |
| NA |  | 9500186100 |  | Bee pollen |  | 4 |
| NA |  | 9500335000 |  | Seaweed |  | 4 |
| NA |  | 3700222501 |  | Milk, human |  | 4 |
| NA |  | 2201073000 |  | Cactus |  | 4 |
| NA |  | 9500275000 |  | Peppermint |  | 4 |
| NA |  | 2001336000 |  | Sesame, seed |  | 4 |
| NA |  | 2405253000 |  | Passionfruit, juice |  | 4 |
| NA |  | 8602000000 |  | Water, indirect, all sources |  | 4 |
| NA |  | 1902105001 |  | Cinnamon-babyfood |  | 4 |
| NA |  | 8601100000 |  | Water, direct, tap |  | 4 |
| NA |  | 9500306000 |  | Psyllium, seed |  | 4 |
| NA |  | 9500218000 |  | Maple, sugar |  | 4 |
| NA |  | 1902274001 |  | Pepper, black and white-babyfood |  | 4 |
| NA |  | 8601200000 |  | Water, direct, bottled |  | 4 |
| NA |  | 802272001 |  | Pepper, nonbell-babyfood |  | 4 |
| NA |  | 9500390000 |  | Vinegar |  | 4 |
| NA |  | 1902274000 |  | Pepper, black and white |  | 4 |
| NA |  | 9500116000 |  | Coffee, instant |  | 4 |
| NA |  | 802272000 |  | Pepper, nonbell |  | 4 |
| NA |  | 2201022000 |  | Bamboo, shoots |  | 4 |
| NA |  | 9500372000 |  | Tea, dried |  | 4 |
|  |  |  |  |  |  |  |
| LAFA, Loss-adjusted Food Availability data series |  |  |  |  |  |  |
| FCID, Food Commodity Intake Database |  |  |  |  |  |  |
| NA, not applicable |  |  |  |  |  |  |
| ^1^Where 1=direct match (cases in which there is no ambiguity about the match, e.g., "apple juice" listed in FCID and LAFA); 2=assumed match (cases in which there is some ambiguity about the match, e.g., "chicken"="chicken liver"); 3=proxy match (cases in which a direct or assumed match was not possible, but a reasonable proxy could be used, e.g., "barley"="buckwheat"; 4=match not possible. | | | | | | |

*Linking NHANES, FCID, and LAFA*

At this point, the linkage between NHANES and FCID has been established (see above), and the linkage between FCID and LAFA has been established (see above), which ultimately allows us to link food intake data (from NHANES) with data on food waste and inedible portions (from LAFA; Supplemental Figure 1). The specific assumptions that were embedded in this process were described above. Yet it bears emphasizing that LAFA provides only a single estimate of food waste and inedible portions for each food, without any variance, and this estimate is consistent across years. Therefore, in the final analysis of this study, any variance in expenditures associated with food waste and inedible portions is due to inter-individual differences in food intake amounts rather than different rates of waste and discards of inedible portions between individuals. This process is imperfect, but does have precedent in previous studies[3, 5-7]. Similarly, since LAFA does not distinguish between food at home (FAH) and food away from home (FAFH), any difference in food waste and inedible portions between FAH and FAFH is due to inter-individual differences in the amount of FAH versus FAFH reported by individuals in NHANES. Furthermore, data are not available to ascertain whether a specific FAH is the leftover portion of a specific FAFH. This could have resulted in mis-categorization bias in these select cases.

**Estimating food prices**

This process links data on the amount of food intake, food waste, and inedible portions (from above) to data on food prices and price inflation, and a final adjustment is made to account for price differences between FAH and FAFH. Parts of this process have precedent in previous studies[8, 9] and were further informed by personal communication with economists at USDA Economic Research Service (USDA ERS), and other parts of this process are novel but reflect recommendations from an expert panel to USDA ERS.[10]

*Linking NHANES with CNPP Food Prices Database*

The USDA Center for Nutrition Policy and Promotion (CNPP) Food Prices Database provides data on the price of each food reported consumed by individuals in NHANES. This linkage between CNPP Food Prices Database and NHANES has been pre-established by staff at USDA ERS, and more details on the construction of this database can be found elsewhere.[11] These data only link to NHANES waves 2001-2002 and 2003-2004, since this data source was discontinued thereafter. In the present study, we only used food price data from 2001-2002 because it covered nearly all foods reported consumed in 2003-2004. Food price data also covered 88% of all foods reported consumed in NHANES from 2005-2016, and the missing food prices (12%) were imputed by averaging the individual food prices in each unique food category (n=41 categories; Supplemental Table 1), weighted by the consumption amount (from NHANES) of each food within each food category. CNPP Food Prices Database does not include prices for alcoholic beverages, so these data were not included in the present study.

*Linking CNPP Food Prices Database with CPI*

To bring food prices (2001-2002) up to date with the most recent food intake data (2015-2016), we consulted with a scientist at USDA ERS (pers. comm., Annemarie Kuhns) to develop methodology to account for food price inflation to 2016. This process utilized the Consumer Price Index (CPI) for each major food group (Supplemental Table 3).[12] The CPI represents the average change in consumer prices over time, and is maintained and published by the US Department of Labor, Bureau of Labor Statistics. This method was previously utilized by others.[8, 9] Data on food price inflation were available for only 15 major food categories (from CPI), whereas data on food intake were available for over 8,500 individual foods (from NHANES), so linking these data may have contributed to over-generalized estimates of contemporary prices for individual foods.

*Linking CNPP Food Prices Database with FoodAPS*

CNPP Food Prices Database provides only retail food prices (FAH prices), so the price of each FAFH was derived using data from the National Household Food Acquisition and Purchase Survey (FoodAPS; Figure 1).[13] This general approach has been suggested by others,[10] but this study represents the first time it has been operationalized, to the best of our knowledge. Using FoodAPS data, we estimated the average FAH and FAFH prices of each major food group, and derived a coefficient that represents the ratio between the price paid for each FAH to the price paid for each FAFH (Supplemental Table 2). This coefficient was then multiplied by the price of each FAFH in the CNPP Food Prices Database to derive its adjusted price. Two major assumptions were embedded in this process. First, given the limitations of hand-coding, it was not feasible to link each food in FoodAPS with each food in CNPP Food Prices Database, so we estimated these coefficients for 11 major food categories. Although approximately 80% of FoodAPS food codes are the same as FNDDS food codes (which link to NHANES), the remaining food codes were manually pre-assigned by FoodAPS staff. Given this constraint, it was only feasible to establish coefficients for major food categories. Further efforts are needed to establish these linkages at the level of food codes rather than food groups. Second, FoodAPS data are only available for 2012-2013. Since these data were only used to estimate the difference in prices between FAH and FAFH, rather than their absolute prices, this means that our final estimates of the relative difference of FAH and FAFH prices reflect differences from 2012-2013. FoodAPS represents the only source of information on FAH versus FAFH prices, so further updates to this database are sorely needed for future research in this domain.

**References**

1. US Department of Agriculture, Economic Research Service (ERS). *Loss-Adjusted Food Availability (LAFA) data series documentation.* 2019. Available at: <https://www.ers.usda.gov/data-products/food-availability-per-capita-data-system/loss-adjusted-food-availability-documentation/> (verified 24 October 2019).

2. US Environmental Protection Agency. *Food Commodity Intake Database (FCID).* 2005-2010. Available at: [http://fcid.foodrisk.org/#](http://fcid.foodrisk.org/) (verified 24 October 2019).

3. Conrad Z, Niles MT, Neher DA, Roy ED, Tichenor NE, Jahns L: Relationship between food waste, diet quality, and environmental sustainability. *PLOS ONE* 2018, 13:e0195405.

4. US Department of Agriculture, Agricultural Research Service: Food and Nutrient Database for Dietary Studies. 2016.

5. Birney CI, Katy FF, Davidson FT, Michael EW: An assessment of individual foodprints attributed to diets and food waste in the United States. *Environmental Research Letters* 2017, 12:105008.

6. Rose D, Heller MC, Willits-Smith AM, Meyer RJ: Carbon footprint of self-selected US diets: nutritional, demographic, and behavioral correlates. *Am J Clin Nutr* 2019, 109:526-534.

7. Heller MC, Willits-Smith A, Meyer R, Keoleian GA, Rose D: Greenhouse gas emissions and energy use associated with production of individual self-selected US diets. *Environ Res Lett* 2018, 13:044004.

8. Rehm CD, Monsivais P, Drewnowski A: Relation between diet cost and Healthy Eating Index 2010 scores among adults in the United States 2007-2010. *Prev Med* 2015, 73:70-75.

9. Rehm CD, Monsivais P, Drewnowski A: The quality and monetary value of diets consumed by adults in the United States. *Am J Clin Nutr* 2011, 94:1333-1339.

10. Muth MK, Birney C, Cuellar A, Finn SM, Freeman M, Galloway JN, Gee I, Gephart J, Jones K, Low L, et al: A systems approach to assessing environmental and economic effects of food loss and waste interventions in the United States. *Sci Total Environ* 2019, 685:1240-1254.

11. Carlson A, Lino M, Juan W, Marcoe K, Bente L, Hiza HAB, Guenther PM, Leibtag E. *Development of the CNPP Food Prices Database,.* Washington, DC: US Department of Agriculture, Center for Nutrition Policy and Promotion, and Economic Research Service;2008. Available at: <https://www.cnpp.usda.gov/data> (verified 28 February 2019).

12. US Department of Agriculture, Economic Research Service. *Consumer Price Index.* 2019. Available at: <http://www.ers.usda.gov/data-products/food-price-outlook.aspx> (verified 24 October 2019).

13. US Department of Agriculture, Economic Research Service. *National Household Food Acquisition and Purchase Survey.* 2012-2013. Available at: <https://www.ers.usda.gov/data-products/foodaps-national-household-food-acquisition-and-purchase-survey/> (verified 24 October 2019).
