# Supplementary material for: Daily cost of consumer food wasted, inedible, and consumed in the United States, 2001–2016
Source: Nutr J. 2020 Apr 20;19:35. doi: 10.1186/s12937-020-00552-w (PMC7168972; doi:10.1186/s12937-020-00552-w)
Supplement: Supplementary file 2 — Additional file 2 : Figure S1. Steps to derive the proportion of food waste from the edible weight of food. Figure adapted, with permission, from Conrad, Zach; Niles, Meredith; Neher, Deb; Roy, Eric; Tichenor, Nicole; Jahns, Lisa. (2018). Relationship between diet quality, food waste, and environmental sustainability. PLoS ONE, 13:e0195405. Text boxes with solid outlines represent data aquired from USDA Loss-adjusted Food Availability data series (LAFA); text boxes with dashed outlines represent derived data. [file 12937_2020_552_MOESM2_ESM.pdf]

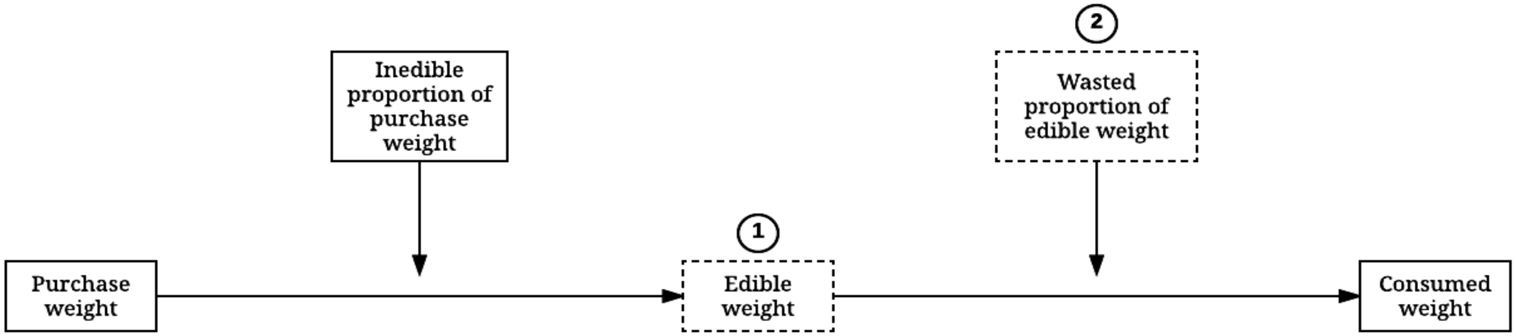

Steps

1. Edible weight = purchase weight x (1-inedible proportion of purchase weight)
2. Wasted proportion of edible weight = (edible weight - consumed weight)/edible weight
